# Recent progress in dendritic pruning of *Drosophila* C4da sensory neurons

**DOI:** 10.1098/rsob.240059

**Published:** 2024-07-24

**Authors:** Menglong Rui

**Affiliations:** ^1^ School of Life Science and Technology, the Key Laboratory of Developmental Genes and Human Disease, Southeast University, Nanjing 210096, People‘s Republic of China

**Keywords:** *Drosophila*, neurodevelopment, c4da sensory neuron, neuronal remodelling

## Abstract

The brain can adapt to changes in the environment through alterations in the number and structure of synapses. During embryonic and early postnatal stages, the synapses in the brain undergo rapid expansion and interconnections to form circuits. However, many of these synaptic connections are redundant or incorrect. Neurite pruning is a conserved process that occurs during both vertebrate and invertebrate development. It requires precise spatiotemporal control of local degradation of cellular components, comprising cytoskeletons and membranes, refines neuronal circuits, and ensures the precise connectivity required for proper function. The *Drosophila*’s class IV dendritic arborization (C4da) sensory neuron has a well-characterized architecture and undergoes dendrite-specific sculpting, making it a valuable model for unravelling the intricate regulatory mechanisms underlie dendritic pruning. In this review, I attempt to provide an overview of the present state of research on dendritic pruning in C4da sensory neurons, as well as potential functional mechanisms in neurodevelopmental disorders.

## 1. Introduction

During animal development, the nervous system builds an elaborate framework, the accurate connectivity of which is essential for its function. Neuronal remodelling is an evolutionarily conserved strategy to accomplish this complex wiring. It is a broadly shared developmental mechanism across the animal kingdom to improve the targeting of dendrites and axons required for the neural circuit’s maturation. In mammals, neuronal remodelling takes place primarily during the early postnatal period and often involves the removal of synapses as well as long stretches of axonal and dendritic branches, sometimes accompanied by the generation of novel connections or the consolidation of existing ones [1–3]. Programmed neuronal pruning belongs to the degenerative part of remodelling, a process that selectively removes inappropriate or exuberant projections that are formed during development without the loss of the parent neuron [2,4,5]. Synapse elimination or dendrite/axon pruning is widely happening in the central nervous system (CNS) and has also been seen in the peripheral nervous system (PNS) [4,5]. An important type of developmental pruning is stereotyped large-scale pruning, in which a substantial fraction of certain primary axonal and dendritic branches is ablated [5]. The precise regulation of neuronal pruning is important for the correct function of the nervous system since defects in pruning have been shown to result in various developmental neurological and psychiatric disorders such as autism spectrum disorder, schizophrenia and Alzheimer’s disease [6–9].

In holometabolous insects, like *Drosophila*, a great number of larval-born neurons are subject to pruning during metamorphosis, a transition phase between larval and adult stages [10,11]. In the CNS of *Drosophila*, mushroom body (MB) γ neurons prune their dorsal and medial axon branches and their whole dendrites [12]. In the PNS, a subset of dorsal dendritic arborization (da) sensory neurons has become an attractive *in vivo* model to investigate the molecular mechanisms underlying dendrite pruning during metamorphosis [4]. The sensory class IV dendritic arborization (C4da) neurons specifically and entirely prune their long and branched larval dendrites through a degenerative form at the beginning of the pupal stage, while leaving their axons intact [11,13]. During development, the class I dendritic arborization (C1da) sensory neurons, which can be divided into ddaD and ddaE, undergo dendrite-specific pruning in the same way as C4da sensory neurons [11,13]. In contrast, class II (ddaB) and class III (ddaA/ddaF) neurons are removed via apoptosis during early metamorphosis [11]. The pruning process consists of both local degeneration and retraction [14], similar to the neurodegenerative changes that occur in cerebral injury and neurodegenerative disorders. Therefore, a thorough study of the cellular and molecular details of developmental pruning would potentially contribute to our comprehension of pathological neurodegeneration associated with injury and neurological disorders. Notably, dendrite remodelling in C4da neurons is not only governed by intrinsic molecular signals, but signals from the surrounding microenvironment or neighbouring cells also influence the process. In the following section, I will discuss in detail the current state of research on the regulatory mechanisms of dendritic pruning in *Drosophila* C4da sensory neurons.

## 2. Ecdysone signalling and its downstream targets

**Figure 1.**
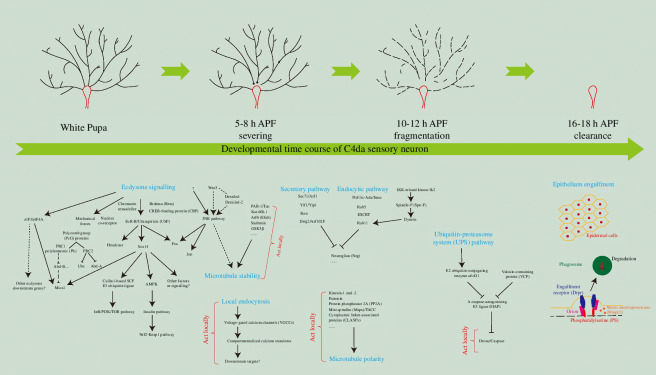
Dendritic pruning of C4da neurons and the related regulatory mechanisms. The schematic representation of the developmental time course of dendritic pruning in C4da neurons and the associated regulatory mechanisms, including ecdysone signalling and its downstream targets, MT stability, MT orientation, the endocytic and secretory pathways, the UPS pathway and local caspase activity and the phagocytosis of epithelial cells. The locally occurring regulatory signals in dendrites are marked in red type. Several representative events are highlighted in blue type.

Multiple important intracellular signals are reported to be involved in the pruning process of dendrites in C4da sensory neurons. Ecdysone signalling is well known as a master switch of developmental remodelling across nervous systems, and the pruning process is initiated by a pulse of the steroid hormone ecdysone at the late larval stage [10]. The neural-specific ecdysone receptor B1 (EcR-B1) has been demonstrated to be cell-autonomously required for the dendritic pruning of the C4da sensory neurons, together with its co-receptor Ultraspiricle (USP) [11,13]. Ecdysone signalling subsequently activates the expression of a series of downstream genes, including the expression of the transcription factor Sox 14 [15]. It is also found that Brahma (Brm)-containing chromatin remodeller and a histone acetyltransferase CREB-binding protein (CBP) cooperate with the steroid hormone ecdysone to regulate Sox14 expression [16]. Next, Sox14 as a transcription factor has been identified to be involved in regulating the expression of Mical, which is an actin depolymerization protein [17]. It shows that the absence of Mical results in impaired dendritic pruning in C4da neurons [15], and thus we understand the classical regulatory signal for dendritic pruning in C4da neurons: ecdysone signalling-Sox14-Mical. Whereas it suggests that the complementation of Mical could only partially ameliorate the dendritic pruning phenotype resulting from the deletion of Sox14 [15], indicating the other downstream target signals of Sox14 in controlling dendritic pruning. Moreover, Cullin1-based SCF E3 ubiquitin ligase has been reported to govern dendritic pruning through the activation of the InR/PI3K/TOR pathway and this process is also verified as one of the downstream branches of Sox14 [18]. In addition, a recent study shows that the evolutionarily conserved Nrf2-Keap1 pathway is activated by steroid hormone signalling to govern neuronal remodelling through the protein degradation pathway [19]. Afterwards, it is found that the metabolic regulator AMP-activated protein kinase (AMPK)-insulin pathway regulates dendritic pruning by activating the Nrf2-Keap1 pathway, a process that also responds to ecdysone signalling [20]. Considering that metamorphosis in *Drosophila* is a non-feeding phase, special metabolic modalities are required to maintain energy supply. Prior research revealed that AMPK maintains the energy requirements for the dendrite pruning process by regulating metabolism [21]. Besides activating Sox14, ecdysone signalling also regulates the expression of Headcase, a cytoplasmic protein of currently unknown function, to promote dendritic pruning in C4da neurons [22] (figure 1). Importantly, more novel target genes or signalling pathways for ecdysone signalling are expected to be discovered in the future.

## 3. JNK pathway, microtubule stabilization and microtubule orientation

The Jun N-terminal Kinase (JNK) pathway is a conserved signalling pathway involved in various cellular processes, including apoptosis and morphogenesis [23,24]. Intriguingly, the JNK signalling has been found to coordinate with ecdysone signalling to facilitate dendrite pruning in C4da neurons via Fos, a component of the AP-1 complex. This pathway functions in a parallel manner to the ecdysone-mediated Sox14 pathway in dendritic pruning. Further studies have shown that Wnt5, a ligand of the non-canonical Wnt signalling pathway, acts genetically upstream of JNK signaling to promote dendrite pruning of C4da neurons. In addition, the related-to-tyrosine-kinase (RYK) receptor Derailed (Drl) and a closely related RYK receptor, Derailed-2 (Drl-2), may serve as a mediator between Wnt5-regulated JNK signalling in dendrite pruning [25]. Given that the JNK pathway can regulate microtubule (MT) stabilization by phosphorylating the MT-binding protein Tau [26,27], it should be a potential mechanism by which the JNK pathway regulates dendritic pruning [25]. This is reasonable because there is considerable evidence that MT plays a crucial role in dendritic pruning. The thinning of proximal dendritic branches is the first morphological sign of dendritic pruning in C4da neurons. Subsequent varicosities formation around the thinned area causes the dendrites to separate from the soma [11,28]. The local collapse of the MTs within the dendrites is a pronounced feature of dendrite pruning in C4da, which happens prior to the membrane dendrites thinning and severing. Previous research has demonstrated that dendritic pruning is inhibited by the deletion of multiple MT-destabilizing proteins, including the Tau kinase PAR−1 [29], the MT-severing enzyme Katanin p60-like 1 (Kat-60L1) [30], the evolutionarily conserved glycogen synthase kinase 3β (GSK3β) [31], and MT-destabilizing factors Arf6 (Efa6)/Stathmin (Stai) [32]. In addition to MT stabilization, recent multiple findings reveal that MT polarity plays an equally critical role in dendrite pruning. In the dendrites of C4da neurons, MTs are oriented almost exclusively minus-end-out in major dendrites, although plus-end-out MTs are present in the terminal branches. However, in axons, MTs are arranged with nearly uniform plus-end-out orientation [33,34]. Interfering with the orientation of MTs in dendrites leads to defective dendrite pruning [35–39] (figure 1). More MT stabilizing and MT polarity-regulating proteins involved in dendritic pruning need to be discovered in the future. Besides MT, dynamic actin changes were also observed during dendritic pruning in C4da neurons [40]. How MT orientation and actin dynamic in dendrites regulate dendritic pruning and whether these mechanisms are conserved in mammals need to be further determined.

## 4. Ubiquitin-proteasome system (UPS) pathway and local caspase activity

**Figure 2.**
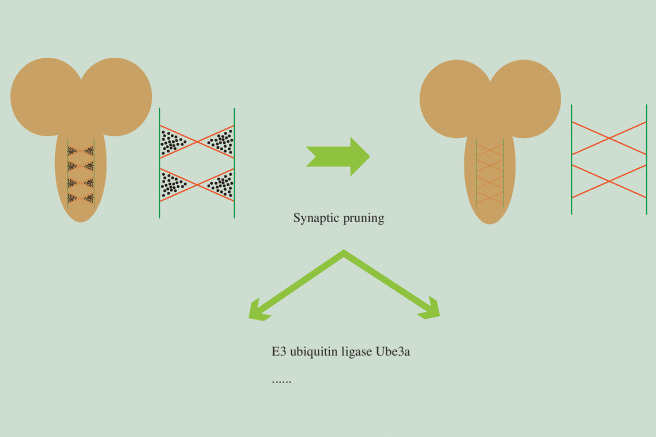
Ube3a E3 ligase is required for synaptic pruning of C4da neurons. The schematic representation of synaptic elimination in the ventral nerve cord (VNC) and the function of Ube3a E3 ligase in synaptic pruning of C4da neurons.

Another well-characterized regulatory mechanism underlies dendritic pruning is the ubiquitin-proteasome system (UPS) pathway-mediated protein degradation. It occurs in a conserved manner in both dendritic and axonal pruning [41,42]. It is found that deletion of E2/E3 ubiquitinating enzymes results in the failure of dendritic pruning. Further studies show that an E2 ubiquitin-conjugating enzyme ubcD1 and a ubiquitin-dependent ATPase Valosin-containing protein (VCP) mediate the degradation of the caspase-antagonizing enzyme DIAP1 [42,43]. This allows for local activation of the Dronc, the homologous protein of Caspase. A study before this has already unravelled that local caspase activity directs the engulfment of dendrites [44]. Collectively, these findings present a mechanistic link between the UPS and apoptotic mechanisms in regulating dendritic pruning. It should be noted that the activity of the UPS pathway during development may also be governed by ecdysone signalling. Additionally, VCP has been discovered to promote dendrite pruning through a regulatory role in mRNA metabolism instead of protein degradation [45]. Thus, unveilling a proteolysis-independent function of the UPS in dendrite pruning (figure 1). Importantly, a creative work by Kazuo Emoto established a novel approach to studying synaptic pruning by investigating the presynaptic marker puncta in the abdominal segments of C4da neurons. They identified that developmental synapse elimination requires the presynaptic activity of E3 ubiquitin ligase Ube3a, which is a causal factor in the developmental disorder Angelman syndrome [46] (figure 2). Noteworthily, this work opens a new avenue for the exploration of synaptic pruning in C4da neurons in the future.

## 5. The endocytic and secretory pathways

Cell adhesion molecules (CAMs) are expressed on the surface of nearly all cells, where they link to the molecules of the extracellular matrix or the receptors of other cells [12]. In addition to playing an important role in the maintenance of cellular structures, CAMs act as signaling receptors, transmitting signals derived via cellular interactions that govern a wide range of processes, such as cell division, migration and differentiation [14,47]. Rab5/ESCRT-mediated endocytic pathways are required for dendrite pruning. Further studies have shown that interference with the endocytic pathway results in a large accumulation of the L1-type CAM Neuroglian (Nrg) on cell membranes and endosomes, while endosomal degradation of Nrg during development is thought to be a key step in initiating dendrite pruning [48]. Intriguingly, pruning defect 1 (*prd1*), a novel gene that physically interacts with the clathrin adaptor protein α-Adaptin (α-Ada) and the kinesin−3 immaculate connections (Imac), facilitates dendrite pruning via the endo-lysosomal degradation of Nrg [49]. Our study subsequently reveals another cell membrane protein, Raw, also plays an important role in dendrite pruning. It regulates dendrite pruning by downregulating Nrg, and this process is dependent on the secretory pathway [50]. Additionally, the loss of the small GTPase Rab11, a regulator of recycling endosomes, causes defects in dendritic pruning [51]. Predictably, a wide array of proteins needs to be degraded during neural pruning, and thus global endocytosis-mediated protein degradation is critical for this process. It is found that local endocytosis in proximal dendrites mediates calcium (Ca^2+^) inward flow, a process that also plays an important role in dendritic pruning [28]. Therefore, it would be interesting to find more key proteins like Nrg that need to be specifically degraded at the beginning of pruning. The protein secretory pathway in cells is essential for the delivery of functional secretory proteins, and disruption of this process leads to a wide variety of severe disorders [5]. Based on previous research, multiple regulators of secretory pathways are involved in the regulation of dendrite pruning in C4da neurons, including Sec71/Arf1 [52], Yif1/Yip1 [53], Droj2/Arf102F [54] and aforementioned Raw [50]. At this point, two fundamental cellular processes of the secretory and endocytic pathways, involving the transport and degradation of proteins, have been identified for their essential functions in dendrite pruning. Interestingly, the regulatory mechanisms of dendritic pruning by all these modulators of the secretory pathway converge on the endocytosis of Nrg (figure 1). How they then maintain the distribution of Nrg on the membrane via the cellular secretory pathway is unclear. It is plausible that there is a yet unknown ligand controlled by the secretory pathway that directly triggers the endocytosis of Nrg. Therefore, it will be interesting to search for the ligand that triggers Nrg endocytosis in future studies. Another possibility is that the secretory pathway directly regulates the protein processing of Nrg, which ultimately promotes Nrg uptake during dendrite pruning.

## 6. Ik2/Spn-F/dynein complex and compartmentalized calcium transients

A previous report suggested that neurons with compromised function of the *Drosophila* IKK-related kinase Ik2 led to an attenuated capacity to sever dendrites of C4da sensory neurons in the puparium [30]. At subsequent, a coiled-coil protein, Spindle-F (Spn-F), functions as a pivotal element connecting Ik2 kinase to the dynein motor, and the generation of the Ik2/Spn-F/dynein complex is essential for Spn-F re-localization and dendritic pruning [55]. A sequential work by Hsiu-Hsiang Lee illustrates how Ik2/Spn-F signalling is transmitted in neurons and eventually leads to the pruning of dendrites. It is the small GTPase Rab11, which can be activated by Ik2/Spn-F signalling, to trigger dendrite pruning in C4da neurons [56]. Additionally, in *Drosophila* C4da neurons, the local increase in endocytic activity helps to delineate dendrites that produce compartmentalized calcium, and these compartmentalized calcium transients in dendrites serve as spatiotemporal signals to induce pruning [28,57] (figure 1). Importantly, in mammalian neurons, the degeneration of axons involves calcium influx through the voltage-gated calcium channels (VGCCs) [58]. Furthermore, both dendrite pruning in C4da neurons and axon degeneration in mouse dorsal root ganglion (DRG) neurons need the co-activation of the two proteases, Caspases and Calpains [57,59]. These results imply an evolutionary conservation of calcium signalling during neural pruning. However, it is still interesting to explore how transient calcium signalling activates downstream factors and thus promotes developmentally relevant axonal and dendritic pruning.

## 7. Polycomb group (PcG) proteins, mechanical forces and translation initiation pathway

Polycomb group (PcG) proteins are key chromatin modulators that keep lineage-inappropriate genes repressed and are thereby essential for the determination of a cell’s destiny [2]. Recent research shows that PcG and Hox genes play a crucial role in modulating ecdysone signaling and dendrite pruning in C4da neurons [60]. Polycomb repressive complex-1 and-2 (PRC1 and PRC2) are two PcG complexes [10,61], the core PRC1 component Polyhomeotic (Ph) selectively promotes Mical expression might partially by silencing the Hox gene *abdominal B* (*abd-B*) [60]. Nevertheless, further research is needed to identify the other downstream target genes of PcG proteins involved in the regulation of dendritic pruning. It is well known that mechanical forces play an active role in shaping cells during development [44]. However, their importance in the morphogenesis of neurons is poorly understood. Sebastian Rumpf’s recent study shows that ecdysone signalling induces the dendrites to be more mechanically fragile. Dendrite severing in the C4da neurons takes place during the period of elevated movements of pupal tissue, which applies mechanical forces to break dendrites. This suggests that mechanical tearing may be a novel mechanism underlying dendrite pruning [62]. It has been found that the axons and proximal regions of dendrites in C4da neurons are encapsulated by peripheral glial cells [63], and thus mechanical dendritic dissection during pruning may be affected by these surrounding cells. Additionally, previous research elucidated that the steroid hormone ecdysone triggers the initiation of dendritic pruning during metamorphosis in *Drosophila* via a translational machinery [15,16]. It is an eIF3-eIF4A-dependent translation initiation pathway that skips 4E-BP to allow sufficient expression of ecdysone downstream genes, including Mical [64] (figure 1). Other translation initiation factors involved in neural pruning await further discovery.

## 8. Phagocytosis of epithelial cells

Previous studies revealed a range of regulatory mechanisms that occur within C4da sensory neurons, so is there a non-cell-autonomous type of regulatory signalling for dendritic pruning? Analogous to physical injury causes neurite degeneration away from the injury site, natural degenerating neurites also need to be clear to preserve tissue homeostasis and avoid inflammatory responses. An innovative study conducted by Yuh-Nung Jan’s laboratory illustrates that in *Drosophila* C4da sensory neurons, epidermal cells are the primary phagocytes responsible for clearing degenerating dendrites during pruning, rather than hemocytes. They further show that epithelial cells engulf and degrade degenerating dendrites via the engulfment receptor Draper (Drpr)-mediated recognition of those ‘labelled dendrites’ [65]. Phosphatidylserine (PS) in neurons acts as a conserved eat-me signal and is recognized by Drpr-expressing phagocytes, mediating phagocytosis of degenerating neurons in *Drosophila*. However, how PS is recognized by Drpr *in vivo* is not well characterized. It has been further elucidated that the chemokine-like protein Orion in *Drosophila* can attach to PS and is capable of sensing PS exposure on neurons. By mediating interactions between PS and Drpr, it facilitates the phagocytosis of fragmented dendrites. In addition, Orion clustering on neurons and phagocytes produces contrasting consequences, and its dosage is a key element in controlling the sensitivity of phagocytes to PS-exposed neurons [66]. Besides the most characteristic phagocytic receptor recognition signaling, whether there are other initiating signals from neighbouring cells involved in governing dendritic pruning in epithelial cells deserves further excavation. Interestingly, a previous literature mentioned that extracellular matrix metalloproteinases (Mmps) activity is required to prune severed larval dendrites during metamorphosis (figure 1). However, cell-intrinsic Mmps are not necessary for this process [13]. These pruning modulators from the extracellular matrix are likely to derive from the surrounding epithelial cells. Thus, future investigations are warranted to shed light on more non-cell-autonomous regulatory signaling in dendrite pruning.

## 9. The PI3K/TORC1 pathway in the regrowth after dendrite pruning

After dendritic pruning, C4da neurons usually regrow novel dendrites to establish functional connections and adapt to the following developmental phase. Owing to the difficulty of experimental manipulation during this stage, the specific mechanisms underlying the regrowth process after dendrite pruning are just starting to be appreciated. A recent study from Rumpf’s lab has suggested that the PI3K/TORC1 pathway orchestrates the regrowth of pruned dendrites by coordinating the activation of protein biosynthesis of regrowth factors including the actin cytoskeleton and exocytosis [67]. Noteworthily, previous studies have also revealed an important role for actin dynamic as well as exocytosis-associated protein secretion process in dendritic pruning [40,50,52,53], suggesting that pruning and regrow share similar regulatory mechanisms. Furthermore, it is conceivable that these two processes are in fact antagonistic events, and that some common mechanisms may influence their course in opposite directions, a notion that needs to be further confirmed. Besides, further exploration of the regulatory signalling underlying dendritic regrowth and its relevance to pruning would be an interesting direction in the future. Understanding these mechanisms not only provides insights into fundamental neurodevelopmental processes but also has implications for addressing neural repair and regeneration in other species, including humans.

## 10. Conclusions and future perspectives

During development, the accurate wiring of the nervous system is the integrated consequence of progressive and regressive events. The selective elimination of unnecessary or superfluous dendrites or axons without resulting in the death of neurons, known as pruning, is a crucial mechanism for maintaining appropriate wiring in the developing nervous system. After decades of work, the molecular basis of dendritic pruning in *Drosophila* has been largely elucidated, although our understanding of this process is still developing. In the future, we can use C4da sensory neurons to further screen for unknown regulatory signals or factors involved in dendritic pruning. In addition, recent work in the Kazuo Emoto’s lab has opened new avenues for future studies of the regulatory mechanisms underlying synaptic pruning using C4da neurons [68]. Considering that numerous previous studies have focused on cell-autonomous regulation, it would be worthwhile to devote more effort in the future to discover the initiating signals of dendritic or axonal pruning by the surrounding microenvironment, such as epithelial cells, glial cells and the extracellular matrix. Importantly, confirming whether these identified mechanisms of dendritic pruning are conserved in mammals will further highlight the significance. It will also be of great interest in the future to investigate whether risk genes of autism, schizophrenia or other related disorders are involved in controlling dendrite or axon pruning. By studying the biological and molecular functions of these genes, the link between abnormalities in neuronal pruning and these neurological disorders can be better understood. Overall, the research on neuronal pruning has far-reaching scientific implications and potential clinical applications for advancing the field of neuroscience, understanding the basics of the nervous system, the pathogenesis of related neurological diseases and developing new therapeutic strategies to treat neurological diseases.

## Data Availability

This article has no additional data.
